# Phosphoproteomic Analysis of Maize Seedlings Provides Insights into the Mechanisms of Heat-Stress Tolerance

**DOI:** 10.3390/ijms26062439

**Published:** 2025-03-09

**Authors:** Zhenyu Ma, Runsi Qi, Huaning Zhang, Xiangzhao Meng, Zihui Liu, Shuonan Duan, Xiulin Guo, Guoliang Li, Zhonglin Shang

**Affiliations:** 1 Institute of Biotechnology and Food Science, Hebei Academy of Agriculture and Forestry Sciences/Hebei Key Laboratory of Plant Genetic Engineering, Shijiazhuang 050051, China; mazhenyuqqtt@163.com (Z.M.); 18633708039@163.com (R.Q.); 13821201341@163.com (H.Z.); xzmeng@haafs.org (X.M.); liuzihui1978@163.com (Z.L.); duanshuonan@haafs.org (S.D.); myhf2002@163.com (X.G.); 2 College of Life Sciences, Hebei Normal University, Shijiazhuang 050024, China

**Keywords:** phosphoproteomics, iTRAQ labeling, heat stress, maize

## Abstract

The dramatically high temperatures triggered by global climate change threaten maize growth and yield. In recent years, increasing attention has focused on the impacts of heat injury on maize. However, the molecular mechanisms behind maize’s adaptation to heat stress remain largely unexplored. To uncover how plants protect themselves from heat stress, we performed a phosphoproteomic analysis on maize leaves by using multiplex iTRAQ-based quantitative proteomic and LC-MS/MS methods. A total of 1594 phosphopeptides ascribed to 875 proteins were identified. A functional enrichment analysis of the proteins and phosphoproteins revealed that the early thermal responses of maize were associated with translational and post-translational modifications, protein turnover, and chaperone binding in the MAPK pathway. A motif analysis also yielded a significant number of potential MAPK substrates. The functional characterization of the phosphoproteins and pathways identified here will provide new insights for improving crop thermal tolerance.

## 1. Introduction

Heat injuries caused by global warming inhibit plant growth and threaten crop yields [1]. Unraveling the signaling mechanisms through which plants protect themselves from heat stress is a critical fundamental question and essential for agricultural sustainability and food security. Maize (*Zea mays*) is a widely produced cereal crop that belongs to the Poaceae family [2]. Recent studies have examined the effects of high temperatures on global food production and revealed that every 1 °C increase in temperature leads to a 7.4% decrease in maize production [3]. In plants, exposure to heat stress (HS) can disrupt photosynthesis, increase the rate of surface transpiration, cause injuries to pollen tubes, and reduce the number of kernels and grain weight during the grain-filling stage [4,5]. At the cellular level, when temperatures exceed a specific threshold, it can disrupt protein folding and membrane fluidity, damage cytoskeletal organization, and interfere with protein trafficking and enzymatic reactions, resulting in metabolic imbalances and the accumulation of harmful substances such as reactive oxygen species (ROS) [6,7,8,9].

In order to survive, plants have developed sophisticated and complex regulatory mechanisms that enable them to resist and adapt to external stresses [10,11]. Heat stress disrupts the fluidity and permeability of the plasma membrane, resulting in increased concentrations of Ca^2+^, ROS, and nitric oxide (NO) in the cytoplasm. These agents consistently stimulate downstream regulatory networks as the secondary messengers [12]. In maize, Ca^2+^ binds to calmodulin (CaM) to form a Ca^2+^-CaM complex in the cytoplasm [13]. This complex integrates the signals transmitted in response to high temperatures, activating the corresponding transcription factors (TFs), such as ZmCAMTA, ZmHsfs, ZmbZIP, ZmMYB-R, and ZmWRKY [14,15,16]. Additionally, the influx of Ca^2+^ into the cytosol activates calcium-dependent protein kinases (CDPKs)—of which there are 35 in maize—as well as calcineurin B-like (CBL) and CIPK proteins, followed by the activation of heat shock transcription factors (HSFs) [17]. Moreover, Ca^2+^ amplifies signals through the activation of downstream genes, such as *HSFA6b*, *ABF1*, *CYCD5;1*, *MutS2*, and heat shock proteins (HSPs), during the reproductive stage via the mitogen-activated protein kinase (MAPK) pathway [16,18].

Protein phosphorylation is a vital and widespread post-translational modification (PTM) that supports the proper functioning of proteins by regulating their conformation, activity, stability, subcellular localization, and interactions [19]. In plants, approximately 47% of the expressed proteins undergo phosphorylation under various conditions [20]. Protein kinases and phosphatases regulate the phosphorylation states of proteins by acting on serine (Ser), threonine (Thr), and tyrosine (Tyr) residues [21]. Several protein kinases catalyze the phosphorylation of substrates. Mitogen-activated protein kinase (MAPK) cascade signal transduction pathways are crucial regulators involved in nearly all fundamental cellular processes [22,23]. A typical MAPK cascade consists of at least three protein kinases. MAPKK (MAPKKK, MKKK, or MEKK) receives signals from sensors, either directly or indirectly, from the external environment, thereby activating its downstream MAPK (MAPKK, MKK, or MEK) and subsequently phosphorylating and activating its upstream MAPK (MPK). The activated MAPKs ultimately phosphorylate downstream transcription factors, cytoskeletal proteins, protein kinases, and other enzymes, leading to the activation of cellular responses [24,25,26].

MAPK cascade signal transduction pathways play significant roles in plant responses to abiotic stress. Research on *Arabidopsis thaliana* has demonstrated that MPK3/MPK6 can phosphorylate the dominant Ser309 of HsfA4A to regulate tolerance to salt stress [27]. In maize, studies on phosphorylation have focused on salt and osmotic stress [28], drought [29], and abscisic acid (ABA)-induced antioxidant activity [30,31]. Notably, 19 MAPK and 74 MAPKKK genes have been identified in maize [32,33]. Research indicates that many MAPK and MAPKK genes are essential for maize’s responses to environmental stresses and phytohormones. Overexpressing *ZmMKK4* in *Arabidopsis thaliana* can improve salt and cold tolerance [34]. Additionally, studies have shown that ZmMKK1 is a positive salt and drought tolerance regulator in transgenic *Arabidopsis thaliana* [35]. *ZmMKK3* is up-regulated by abscisic acid to participate in ABA signaling [36]. Early research revealed that ZmMPK5 [37] and ZmMPK4 [38] are significant in responses to cold stress. Overexpression of *ZmMAPK1* in *Arabidopsis thaliana* can enhance drought tolerance and heat tolerance in plants [39]. ZmMPK3-1 and ZmMPK6-1 are crucial in nutrient deficiencies and heavy metal stresses in maize roots [40]. Extensive research has been conducted on the MAPK pathway and members of the MAPK family. However, the phosphorylation of proteins under heat stress and the MAPK pathways have yet to be systematically studied in maize during heat stress. In this paper, we aim to analyze the impacts of heat stress on the MAPK pathway using phosphoproteomics methods and identify phosphoproteins.

## 2. Results

### 2.1. Proteome and Phosphoproteome Analyses of Maize Seedlings Under Heat Stress

To study heat-responsive proteins and phosphopeptides in maize, we used TiO_2_-enriched phosphopeptide technology alongside label-free quantitative proteomics methods to analyze maize seedlings’ overall proteome and phosphoproteome.

**Figure 1:**
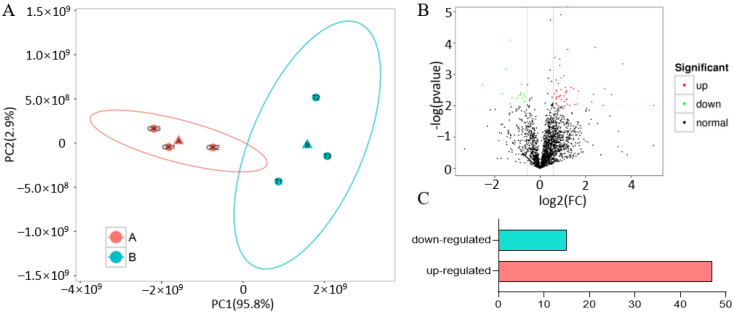
Principal component analysis (PCA) and differential expression protein analysis: (**A**) PCA plots displaying the repeatability and heterogeneity of sequencing samples; (**B**) a volcano plot showcasing the proteins’ differential expression, with significantly upregulated proteins shown in red and downregulated proteins in green; (**C**) counts of differentially expressed proteins.

We performed a principal component analysis (PCA) based on the FPKM values of all the expressed proteins to assess the repeatability and heterogeneity of sequencing samples. As anticipated, the three biological replicates of the CK and Treatment clustered together, indicating strong reproducibility among these biological replicates. Additionally, the samples were grouped into two categories on the principal component PC1, suggesting that heat stress was the main factor causing the differences in protein expression between the CK and Treatment sample (Figure 1A). In the total proteome, we identified 9333 peptides and 2768 proteins across the six samples, each consisting of three biological replicates from the CK and Treatment, with proteins that had missing values omitted (*p* ≤ 0.05, DFR ≥ 1.2) (Table S1). This finding suggests that the repeatability of the sequenced samples is valid. In the phosphoproteome analysis, we identified 1594 phosphopeptides linked to 875 proteins (defined as phosphopeptide isoforms in Proteome Discoverer 2.4). The proteins that were significantly upregulated and downregulated in maize following heat stress are presented in Figure 1B. There were 62 proteins with different expressions, with 47 proteins upregulated and 15 proteins downregulated at the protein level (Figure 1C).

### 2.2. Identification of Phosphorylation Motifs Responding to Stresses

**Figure 2:**
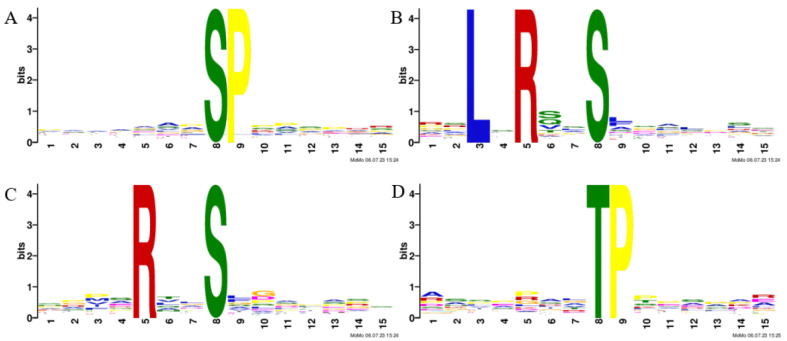
Motif analysis of heat-responsive phosphopeptides: (**A**) the SP motif showed significant enrichment; (**B**) the LxRxxpS motif exhibited significant enrichment; (**C**) the RxxpS motif demonstrated significant enrichment; (**D**) the TP motif revealed significant enrichment.

A motif analysis was conducted on the heat-responsive phosphosites to predict the associated kinases. Four motifs were significantly enriched from the upregulated phosphopeptides due to heat. A serine-directed analysis revealed that the SP motif (Figure 2A) was enriched considerably; this motif is extremely common and a potential substrate for MAPK and calcium-dependent protein kinase (CDPK). The LxRxxpS and RxxpS motifs enriched through the serine-direction analysis (Figure 2B,C) were targeted by SNF1-related kinase II (SnRK2s), SNF1-related kinase III (SnRK3s), and calmodulin-dependent protein kinase (CaMK). In the threonine-directed analysis, we enriched the TP motif (Figure 2D), an extremely common motif and a potential substrate for MAPK [41].

### 2.3. Functional Analysis of the Heat-Responsive Proteome and Phosphoproteome Reveals Different Cluster of Orthologous Groups of Proteins

**Figure 3:**
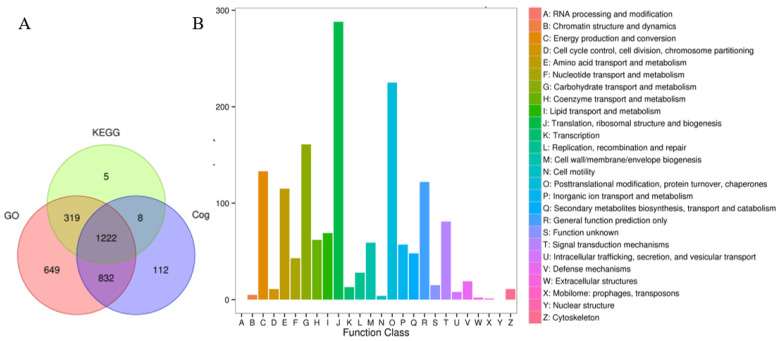
Statistics on protein quantity and functional annotation. (**A**) Venn diagram illustrating the statistical results of protein functional annotations. GO, KEGG, and COG represent three annotated databases. (**B**) COG function classification analysis of all proteins. A–Z stand for 26 different categories.

To investigate protein function, assess the significance of proteins, and explore biological processes such as protein networks and signal transduction pathways, we identified proteins functionally through an alignment analysis using the GO, KEGG, and COG databases. A total of 2504, 1414, and 1364 proteins were annotated from these three databases, respectively. Additionally, 1222 proteins were found concurrently in all three databases (Figure 3A). In Figure 3B, most proteins were categorized into Class J (translation, ribosomal structure, and biogenesis) and Class O (post-translational modification, protein turnover, and chaperones) based on the analysis of the COG (Cluster of Orthologous Groups of proteins) database.

### 2.4. Gene Ontology Analysis of the Heat-Responsive Proteome and Phosphoproteome

**Figure 4:**
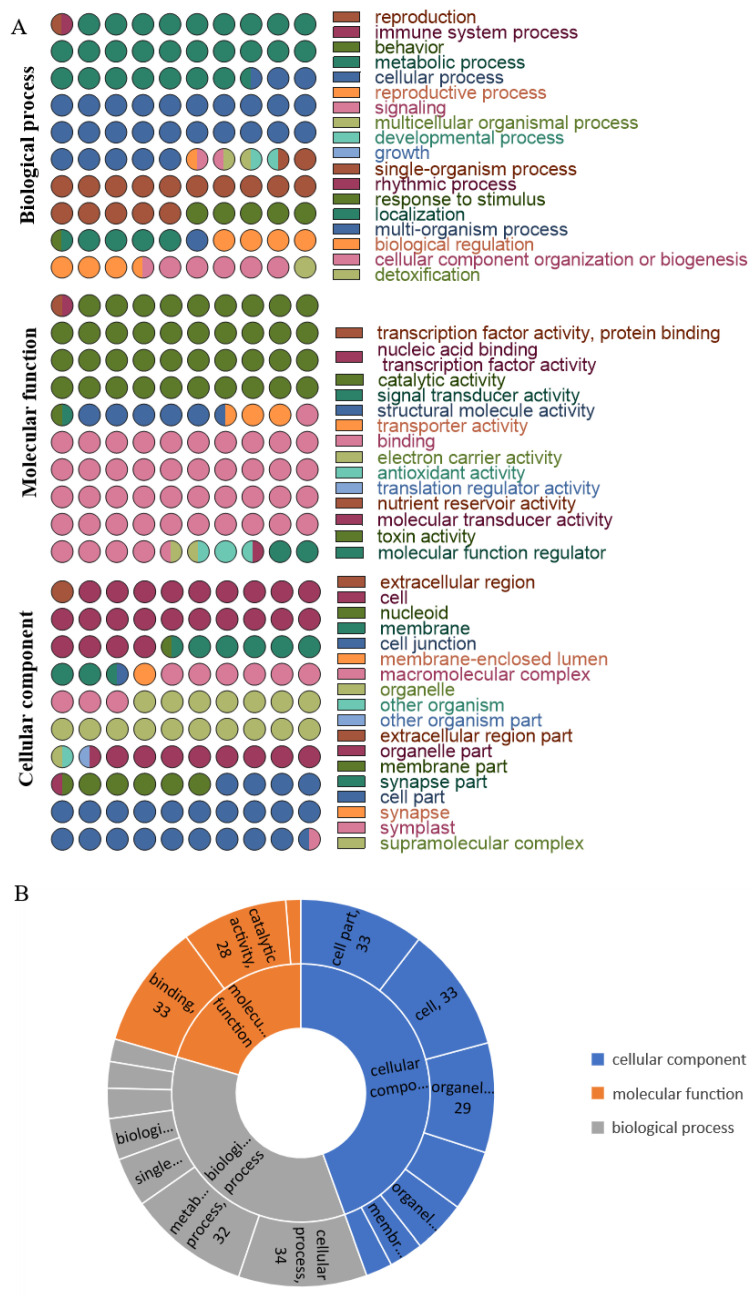
GO functional annotations of total proteins and differential expression proteins. (**A**) GO analysis of all identified proteins. The various colors represent different GO classes, and the number of dots indicates how many proteins were enriched in each class. (**B**) GO enrichment analysis of different expressed proteins. The numbers show how many proteins were enriched in each class.

We conducted gene ontology (GO)-based functional annotation analyses of the heat-responsive proteome and phosphorylated proteome using the protein expression profile and phosphorescence profile. All identified proteins were categorized into three functional categories: biological process (BP), cellular component (CC), and molecular function (MF). The GO BP enrichment analysis revealed that the predominant biological processes were cellular processes, metabolic processes, and single-organism processes. The GO MF enrichment analysis indicated that the most common molecular function was binding and catalytic activity. In the GO CC clustering, the analysis results demonstrated that most proteins were enriched into the cell and cell part subclasses (Figure 4A).

Different GO functional terms within the protein group display significant variations. Specifically, under heat-stress conditions, the proteins enriched in GO-BP group demonstrate notable expression in cellular function regulation and management processes. In contrast, the GO-MF group shows increased biological activities, including interactions and catalytic functions. Importantly, this functional term does not include antioxidant or nutrient reservoir activities. The GO-CC analysis significantly expresses the enriched proteins across various cellular regions and components. However, these regions have not exhibited dynamic protein changes in the extracellular environment (Figure 4B).

### 2.5. Kyoto Encyclopedia of Genes and Genomes (KEGG) Pathway Enrichment Analysis Revealed Thermally Responsive MAPK Signaling Pathway Proteins

**Figure 5:**
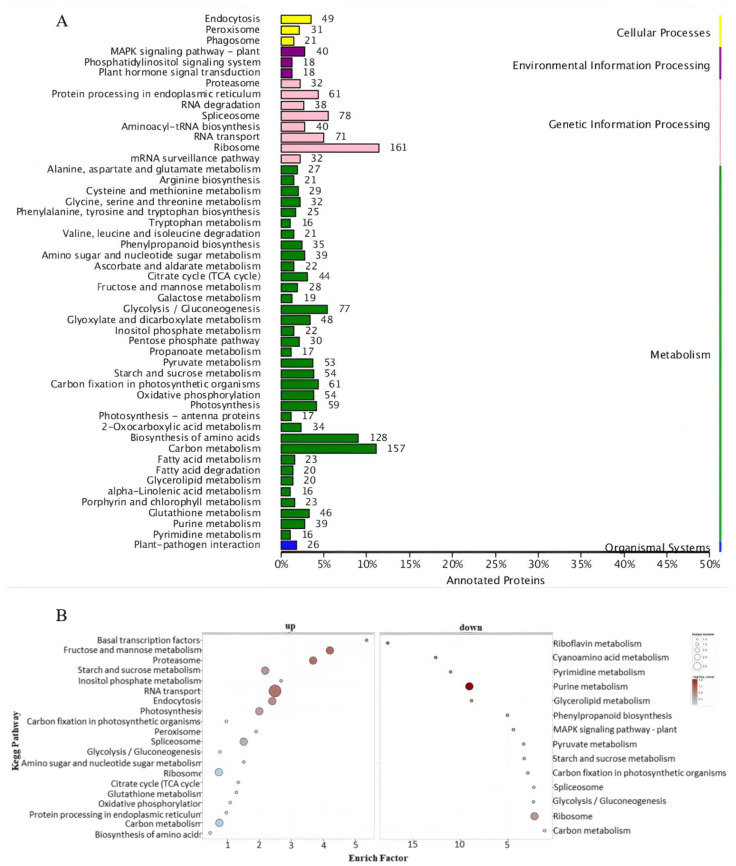
KEGG pathway enrichment analysis: (**A**) KEGG pathway enrichment analysis of all proteins; (**B**) functional enrichment analysis of upregulated and downregulated expressed proteins following heat stress.

The KEGG pathway enrichment analysis indicated that all identified proteins grouped into five categories: cellular processes, environmental information processing, genetic information processing, metabolism, and organismal systems. Proteins within each cluster were enriched in distinct pathways. The metabolism pathway was abundant in various proteins divided into several subpathways. There were three pathways enriched in environmental information processing (Figure 5A).

The environmental information processing cluster includes the MAPK signaling pathway—plant, phosphatidylinositol, and plant hormone signal transduction, as illustrated in Figure 5A. Mitogen-activated protein kinase (MAPK) cascades serve as essential signaling modules downstream of receptors and sensors, which can detect both endogenously produced stimuli and exogenously originated stimuli, such as environmental factors [23]. The MAPK signaling pathway in plants comprises the largest number of pathways, totaling forty (Table S2). Among these forty components, we identified two mitogen-activated proteins (A0A1D6NVH8 and B4FTG6). Within this MAPK signaling pathway, there were two pyrabactin resistance-like proteins (B4FD84 and K7WBY4) that play a crucial role in elucidating the signal transduction of ABA and its regulatory mechanisms. We also identified a pathogenesis-related protein (Q19VG6), a nucleotide diphosphate kinase (C0HHC4), and a bZIP transcription factor (A0A1D6EHU0) from the MAPK signaling pathway.

The KEGG pathway enrichment analysis also revealed that the upregulated proteins were involved in 20 pathways, while the downregulated proteins participated in 14 pathways. The family of phosphatidylinositol transfer proteins, which are enriched in several upregulated pathways, can carry out various biological functions at different stages of plant life [42,43]. The KEGG pathway analysis showed that the MAPK signaling pathway was enriched in the downregulated proteins (Figure 5B).

### 2.6. Identification of Proteins Response to Heat Stress in Proteome and Phosphoproteome

In this study, a total of twenty-three Hsps were identified. There are five members of the Hsp90 protein family, two of which are Hsp70–Hsp90 organizing protein 3. The Hsp70 proteins were the most abundant, with eleven identified in total. These include Heat shock 70 kDa protein 14, Heat shock 70 kDa protein 6, and Heat shock 70 kDa protein 3. Additionally, four small molecular Hsps were identified (Table 1).

Heat-stress-responsive transcription factors have been most widely studied in plants. To thoroughly investigate heat-stress-responsive transcription factors, the protein sequences were extracted and analyzed using iTAK tools (http://itak.feilab.net/cgi-bin/itak/index.cgi) to predict transcription factors. Furthermore, transcription factors from other families, such as bZIP, TCP, bHLH, and MYB, were also identified (Table 2).

## 3. Discussion

In recent years, the frequent occurrence of extreme heat stress threatens global food security and biodiversity. Maize, an important cereal crop worldwide, also suffers from high temperatures. Recent studies have underscored the growing recognition of thermal signaling pathways in plant biology. Protein phosphorylation in plant cells may play regulatory roles in signaling environmental risks. This study utilized TiO2-enriched phosphorylated peptide technology along with label-free quantitative proteomic methods to thoroughly analyze changes in protein phosphorylation in maize seedlings subjected to high-temperature conditions. The results indicated that heat stress significantly and complexly affects maize’s biological activity. This research offers a new perspective on understanding the mechanisms behind corn’s tolerance to high temperatures, providing significant scientific value and practical application prospects.

A motif analysis was conducted to detect phosphorylation motifs associated with specific kinases under stress conditions. Previous research on *Arabidopsis thaliana* has shown that the RXXS motif is a common target of calcium-dependent protein kinase (CDPK) [41,44]. In this study, a total of 9333 peptides and 2768 proteins were identified in the proteome (Table S1). The serine-directed analysis indicated that the SP motif was highly enriched and was a potential substrate for MAPK and CDPK. The serine-directed analysis also identified significant associations with the LxRxxpS and RxxpS motifs, primarily recognized by SnRK2s and SnRK3s. The threonine-directed analysis detected the TP motif, a common substrate for MAPK, CDPK, and SnRK2. Our results are consistent with previous studies on *Arabidopsis thaliana.*

Heat stress impacts plant physiological processes, damages cellular membranes, inhibits chlorophyll synthesis, and causes protein degradation. At the cytological level, heat stress can alter the fluidity of cellular phospholipid membranes, leading to changes in their structural conformation and activity, resulting in a significant reduction in ion exchange. This process involves various components, including second messengers (Ca^2+^ and ROS), gene expression, and protein homeostasis [12]. Ca^2+^ channels such as AtCNGC6, OsCNGC14, and OsCNGC 16 mediate Ca^2+^ signaling triggered by heat stress [45]. AtCaM3 acts as a Ca^2+^ sensor to enhance thermotolerance. The Gene Ontology analysis identified several proteins involved in the transduction and regulation of Ca^2+^ signaling and calcium-dependent lipid binding. Two proteins (P41040 and Q43699) were enriched in the MAPK signaling pathway. Consequently, changes in metabolic responses and cellular adaptations due to abiotic stress are likely attributed to adjustments in response to heat stress. Heat stress induces dynamic processes such as regulating biological functions and metabolic activities associated with oxygen consumption. This finding provides significant insights into understanding the mechanisms of protein modification in dynamic biological processes.

The KEGG enrichment analysis results were consistent with the GO analysis. Most proteins are enriched in metabolism, environmental information processing, and genetic information processing. Our findings suggest that the MAPK signaling pathway plays a significant role in environmental signaling. For instance, this pathway includes one MAPKK (ZmMKK2, A0A1D6NVH8) and one MAPK (ZmMPK7, B4FTG6) (Table S2). In *Arabidopsis*, MKK2, part of the group A MAPKKs, has been reported to be upstream of MPK4 and/or MPK6 in the plant’s response to cold and salinity [46,47,48]. The MEKK1-MKK1/MKK2-MPK4/MPK11 cascades are essential for pattern-triggered immunity (PTI) and effector-triggered immunity (ETI) [49]. However, there has been no detailed report on ZmMKK2 in maize. Research has indicated that ZmMPK7 can eliminate ROS in response to ABA and H_2_O_2_ [50]. Furthermore, increased activation of ZmMPK7 is associated with SA-regulated broad-spectrum resistance to biotic stresses [51].

It is well known that numerous transcription factors (TFs) and proteins play a role in plant response to heat stress. In this work, several Hsp90 (A0A1D6LMW9 and A0A1D6QK75), Hsp70 (A0A1D6FN98 and A0A804QPJ4, B7ZZ42), and small molecular heat shock proteins (HSPs) (A0A804P9D2 and B4FES7) were identified. In maize, heat-stress signaling initiates with physical change at the plasma membrane. This is followed by the rapid influx of Ca^2+^ through calcium channels, CNGC, and glutamate heat receptor-like channels into the cytoplasm [9]. Heat-stress signals are conveyed in the cytoplasm by activating TFs and HSPs [52]. *ZmHsf-06* was upregulated under heat stress [53], with its protein interacting with Hsp70-2 and Hsp70-4, significantly contributing to abiotic stress response. Previous studies have indicated that ZmsHSP26, ZmHSP68, ZmHSP70, ZmHSP90, and ZmHSP101 are crucial for maize’s response to heat stress [54]. Additionally, bZIPs, proteins in the MAPK signaling cascade, were identified in this study. We also identified two bZIPs (A0A1D6EHU0 and A0A1D6MZQ6) involved in the MAPK signaling pathway and several TFs, such as TCP, bHLH, and MYB.

The results demonstrated comprehensive proteome and phosphoproteome profiling of the molecular events in maize seedling under heat stress, providing a much-needed resource for uncovering the molecular basis of heat adaptation in maize.

## 4. Materials and Methods

### 4.1. Plant Materials and Growth Conditions

Maize inbred variety “H21 (♀ Huangzao 4 × H84 ♂)” was used for this study. Maize inbred variety “H21” is one of the primary inbred lines in China and is widely used as a parent in breeding hybrids. The plants were grown in large pots filled with nutrient-rich soil in a greenhouse. When the seedlings grew to having two leaves, some plants were subjected to HS at 42 °C for 30 min (Treatment). The plants grown under normal conditions were CK. The second leaves were sampled quickly, frozen in liquid nitrogen, and stored until protein extraction.

### 4.2. Protein Extraction and Digestion

Proteins were extracted from frozen tissues using a modified phenol–methanol protocol. The proteins were dissolved in 300 μL of 8 M urea and incubated with a 10% concentration of protease inhibitor relative to the lysate. After centrifuging at 14,100× *g* for 20 min, the supernatant was collected as an extract. The concentration of the protein solution was measured using the Bradford assay and then diluted to a final concentration containing an aliquot of 50 μg protein from each sample. This extract was reduced with a 200 mM dithiothreitol (DTT) solution at 37 °C for one hour. The resulting extract was further diluted eightfold by adding 50 mM of ammonium bicarbonate (ABC) buffer. Then, trypsin (trypsin: protein = 1:25) was added, and the solution was incubated at 37 °C overnight.

### 4.3. Phosphopeptide Enrichment and Identification

Phosphopeptides were enriched using TiO2-tips following the manufacturer’s protocol. The phosphopeptides were reconstituted in 0.1% formic acid (FA) for nano LC-MS analysis. Label-free mass spectrometry was performed with a Thermo Orbitrap Fusion mass spectrometer. The scan events were configured as a full MS scan of 250–1450 *m*/*z* at a mass resolution of 120,000, followed by a CID MS/MS scan repeated on the 20 most intense ions selected from the previous full MS scan, along with an isolation window. MS/MS spectra were searched against the Uniprot_*Zea_mays* database (downloaded 14 June 2013). The resulting MS/MS data were processed using Maxquant 1.5.2.8. The remaining peptides were lyophilized for phosphopeptide enrichment.

Protein identification was performed as follows: precursor ion mass tolerance of ±15 ppm; fragment ion mass tolerance of ±0.02 Da; maximum missed cleavages set to 2; static modification of carboxyamidomethylation (57.021 Da) for Cys residues; and dynamic modifications involving oxidation (+15.995 Da) for Met residues. Based on the *p*-value of primary data, data with *p* ≤ 0.05 and a difference ratio ≥ 1.2 were selected for further analysis.

### 4.4. LC-MS/MS Data Analysis and Bioinformatics Analysis

The PCA analysis was performed using the R software (version 3.1.1) and visualized with the ggplot2 package within the R environment [55]. The log2-transformed intensity values of all proteins and phosphopeptides shared among the six samples were used as the features for the PCA. The GO and KEGG enrichment analyses were performed on proteins or phosphopeptides using ClusterProfiler in the R environment, with an FDR < 0.05 indicative of over-representative terms. GO annotations were sourced from the UniProt database (release of May 2021). KEGG pathway mapping of maize proteins was performed online with (http://www.kegg.jp/kegg/pathway.html). For the motif analysis, 15 bp amino acid sequences centered on the phosphosites were submitted to MoMo (Modification Motifs, https://meme-suite.org/meme/tools/momo) and processed using the following settings: width = 15, minimum number = 20, and *p*-value threshold = 0.000001. Venn diagrams were plotted using the ggplot2 R packages.

## Figures and Tables

**Table 1:** Heat stress response protein (at the protein abundance levels).

Accession	*p*-Value	Ratio	Annotation
C0P732	0.1284	0.51	Hsp70-Hsp90 organizing protein 3
C4J4W3	0.1857	1.26	Hsp70-Hsp90 organizing protein 3
A0A1D6ES19	0.1334	0.54	Heat shock protein 90-2
A0A1D6LMW9	0.7631	1.04	Endoplasmin-like protein
A0A1D6QK75	0.5398	1.12	Heat shock protein 90-5 chloroplastic
C0P4Q3	0.2017	0.58	Heat shock protein 90
C3UZ63	0.4041	0.89	HSP protein
A0A804P5Q4	0.0821	1.59	Hsp70-interacting protein N-terminal domain-containing protein
A0A096R6Z8	0.2308	1.24	Heat shock 70 kDa protein 6 chloroplastic
A0A1D6FN98	0.6692	1.43	Heat shock 70 kDa protein 14
A0A1D6LZY7	0.3093	1.24	Heat shock protein 4
A0A1D6MUE8	0.9980	1.00	Heat shock 70 kDa
A0A1D6N7I4	0.1511	1.26	Putative mediator of RNA polymerase II transcription subunit 37c
A0A804PDW7	0.1129	1.62	Hsp70 protein
A0A804QPJ4	0.2531	1.34	Heat shock 70 kDa protein 14
A0A804U6K7	0.8292	0.95	Heat shock 70 kDa protein, mitochondrial
B7ZZ42	0.0848	1.22	Heat shock 70 kDa protein 3
O24581	0.3969	1.14	Luminal-binding protein 3
A0A1D6KC46	0.0483	1.34	Hsp20/alpha crystallin family protein
A0A804LS48	0.7771	1.14	SHSP domain-containing protein
A0A804N0A1	0.8155	1.20	SHSP domain-containing protein
A0A804P9D2	0.0892	3.34	SHSP domain-containing protein
B4FES7	0.4068	2.74	SHSP domain-containing protein

Note: “Ratio” refers to protein abundance ratio of treated/control; “*p*-value” refers to Benjamini–Hochberg adjusted *p*-value.

**Table 2:** Heat-responsive transcription factors (at the protein abundance levels).

Accession	*p*-Value	Ratio	Annotation
A0A1D6LVA4	0.1776	2.23	Putative bZIP transcription factor superfamily protein
A0A804QAV4	0.0240	1.38	bZIP domain-containing protein
A0A060CZ75	0.6253	1.17	bZIP transcription factor (Fragment)
A0A1D6F077	0.5084	0.52	Zinc finger protein 1
B6T7E5	0.0011	2.06	Transcription initiation factor IIF subunit alpha
A0A317YGE4	0.6658	0.93	Transcription initiation factor TFIID subunit 8
A0A1D6M0J7	0.9827	1.00	Transcription factor IIA alpha/beta subunit
A0A804U6V2	0.2892	1.22	HTH TFE/IIEalpha-type domain-containing protein
A0A060CYI1	0.1889	1.47	TCP transcription factor (Fragment)
A0A804P0E2	0.0202	1.86	DM2 domain-containing protein
A0A804NI83	0.8284	1.04	RWD domain-containing protein
A0A804QSX8	0.9783	1.00	[RNA-polymerase]-subunit kinase
A0A096SVP6	0.1394	1.28	Octicosapeptide/Phox/Bem1p family protein
B6TWP6	0.7919	0.97	PB1 domain containing protein
B7ZY51	0.4473	1.33	Octicosapeptide/Phox/Bem1p family protein
A0A804LLP1	0.5643	1.93	NAD(P)-binding domain-containing protein
A0A1D6GVJ8	0.1148	0.74	Myb family transcription factor PHL6
A0A1D6KQT7	0.6926	1.06	Trihelix transcription factor GT-2
B4FI06	0.0266	1.47	MBF1 transcription factor
B6T6H6	0.1976	1.99	bHLH transcription factor
A0A1D6EHU0	0.4666	1.36	Transcription factor VIP1
A0A1D6KKX7	0.2640	1.33	DNA binding protein

Note: “Ratio” refers to protein abundance ratio of treated/control; “*p*-value” refers to Benjamini–Hochberg adjusted *p*-value.

## Data Availability

Data are contained within the article and Supplementary Materials.

## Supplementary Materials

The following supporting information can be downloaded at https://www.mdpi.com/article/10.3390/ijms26062439/s1.
