# Supplementary material for: Phosphoproteomic Analysis of Maize Seedlings Provides Insights into the Mechanisms of Heat-Stress Tolerance
Source: Int J Mol Sci. 2025 Mar 9;26(6):2439. doi: 10.3390/ijms26062439 (PMC11942004; doi:10.3390/ijms26062439)
Supplement: Supplementary file 1 [file ijms-26-02439-s001.zip › ijms-3421619-supplementary/附表/Supplementary Table S2. Heat responsive protein in MAPK pathyway.pdf]

Table S2 Heat responsive protein in MAPK pathyway.

Accession	*p*-Value	Ratio	Annotation
A0A1D6EHU0	0.4666	1.36	bZIP-transcription factor 23
A0A1D6NVH8	0.0222	1.61	mkk2-mitogen-activated protein kinase kinase2
B4FD84	0.0956	1.45	pyl13-pyrabactin resistance-like protein13
B4FTG6	0.0973	0.62	MAPK signaling pathway-plant
B6UHU1	0.3817	1.31	cat1-catalase1
C0HHC4	0.8240	0.97	ndk1-nucleotide diphosphate kinase1
K7V0F9	0.1491	1.42	rboh13-respiratory burst oxidase13
K7WBY4	0.4024	1.20	pyl14-pyrabactin resistance-like protein14
P41040	0.1721	1.40	cal1-calmodulin1
Q19VG6	0.7328	1.43	prp2-pathogenesis-related protein2

Note: “Ratio”refers to protein abundance ratio of treated/control. “*p*-value” refers Benjamini–Hochberg adjusted *p*-value.
